# NIH-funded neonatologist physician-scientists: an exploration of equity and success

**DOI:** 10.1038/s41390-025-04224-5

**Published:** 2025-06-30

**Authors:** Eric N. Horowitz, Misty Good, Kerri Z. Machut, Kaashif Ahmad, Clyde J. Wright, Krithika Lingappan

**Affiliations:** 1 Department of Newborn Medicine, Boston Children’s Hospital, Boston, MA USA; 2 Division of Neonatal-Perinatal Medicine, Department of Pediatrics, University of North Carolina at Chapel Hill School of Medicine, Chapel Hill, NC USA; 3 Division of Neonatology, Northwestern University Feinberg School of Medicine, Chicago, IL USA; 4 Pediatrix Center for Research, Education, Quality and Safety, Sunrise, FL USA; 5 Section of Neonatology, University of Colorado Anschutz Medical Campus, Aurora, CO USA; 6 Division of Neonatology, Department of Pediatrics, Children’s Hospital of Philadelphia, Philadelphia, PA USA

## Abstract

**Background:**

The physician-scientist workforce in pediatric subspecialties is stagnant, with few trainees choosing a research-intensive career path. Limited data are available on the physician-scientist workforce in neonatal-perinatal medicine.

**Methods:**

We performed an anonymous national survey among NIH-funded physician-scientists in neonatal-perinatal medicine with a response rate of 53%. The survey included questions measuring funding, organizational support, personal and professional obligations, academic accomplishments, and career satisfaction.

**Results:**

Differences within gender identity, career stage, international medical graduate status, or underrepresented in medicine were compared. Among these groupings, we found gender identity to be the most common factor associated with significant differences. Among these, the total number of publications for women was lower at 23 versus 88 (*p* = 0.014) for mid-career and 70 versus 185 (*p* = 0.014) for late-career women compared to men. Women more commonly reported periods of time-off from their careers (43% versus 13%, *p* = 0.005), with childbirth being the most common reason. Most respondents reported satisfaction with their career as a physician-scientist. Greater concerns for balance between personal and professional time and increases in multiple measures of burnout were reported by women physician-scientists.

**Conclusion:**

These results identify additional actions to promote entry into and success along the neonatologist physician-scientist career pathway.

**Impact:**

- This is the first paper to examine, with high granularity, the professional and personal characteristics of NIH-funded physician-scientists in neonatology.
- This study identifies areas where professional experiences differ from professional group recommendations and where additional equity work may benefit those along the physician-scientist career pathway.
- By understanding NIH-funded neonatologists’ professional experiences and accomplishments, efforts to foster the success of all those following this career path can be better informed.

## Introduction

Peer-reviewed, conflict-disclosed, independent, and rigorous research leading to scientific discovery is a critical component for the evolution of healthcare. Among those seeking to achieve this is the physician-scientist. The role of a physician-scientist is multifaceted, encompassing clinical care, research, and education. Physician-scientists help to bridge the gap between clinical practice and research by leveraging their clinical insights to inform and guide scientific investigations and produce discoveries that improve clinical care. Physician-scientists in neonatology engage in research to advance our understanding of neonatal diseases, develop new therapeutic strategies, and improve healthcare delivery. Physician-scientists also play a critical role in training the next generation of neonatologists, mentoring fellows and junior faculty while fostering a nurturing environment for scholarly activity and research. This mentorship is essential for sustaining the physician-scientist training pathway in neonatology, especially given the challenges of balancing clinical duties with research.

Institutional and grant support are critical to foster the success of physician-scientists. However, administrative and academic leaders face distinct challenges in assuring the needed support to establish or sustain robust research programs. These challenges stem from balancing the financial constraints imposed by current funding models, the external funding environment, and the substantial long-term investments required to develop and support physician-scientist careers.^1,2^

The physician-scientist workforce in neonatology is declining, with fewer trainees choosing a research-intensive career path. The number of neonatologists who devote at least 50% of their time to research has decreased from 4.5% in 2009 to 3.1% in 2019, making neonatology the fourth lowest across the 14 pediatric subspecialties.^3^ Several factors likely contribute to this decrease in neonatologists choosing a research-intensive career. Attaining and maintaining NIH funding is challenging, and appropriate support at different career stages is crucial for success. Further, it has been reported that NIH awards for pediatric research appear to be disproportionately awarded to a select group of institutions.^4^ This can compound funding challenges for those seeking research success at other institutions.

Data regarding the impact of innate and organizational factors on the physician-scientist workforce in neonatal-perinatal medicine is limited. To expand our knowledge, we conducted a national survey focused on a subset of scientific investigators, the NIH-funded neonatologists. The survey sought to explore their personal characteristics, experienced institutional support, professional duties, accomplishments, and burnout. These results were then used to analyze how these factors may change at the intersection of personal identities and professional expectations. This knowledge can help inform further actions to strengthen the physician-scientist pathway, help recruit the next generation, and sustain the current workforce.

## Methods

To identify NIH-funded neonatologists in the United States, we queried the NIH RePORTER (https://projectreporter.nih.gov/reporter.cfm) on March 15, 2021, using the following search criteria: Department type: “Pediatrics” and Activity code: “R01 Equivalents” and “Research Career Awards” for active grants. An additional search was conducted using “Children’s Hospital” in the “Organization” field with the qualifier set to “contains.” The resulting list of grant awards was exported from NIH RePORTER and curated to remove duplicates. Those holding multiple awards were counted only once. Among this group, we identified principal investigators (PI) who were board-eligible or board-certified neonatologists. We thus identified our cohort of NIH-funded neonatologist physician-scientists (*n* = 131), and their contact emails were determined via an internet search.

An anonymous 70-item REDCap survey was developed for this study and was distributed by email from March through April 2021 (one year into the COVID-19 pandemic) to all identified NIH-funded neonatologists. The survey included questions from our previous work and examined gender identity, demographic variables, organizational support, funding, personal and professional obligations, academic accomplishments, and career satisfaction.^5^

Within organizational support, new questions were asked about experiences during their time as an early-stage investigator. Per NIH, an early-stage investigator is a principal investigator (PI) who has completed their terminal research degree or end of post-graduate clinical training, whichever is later, within the past ten years and who has not previously competed successfully as a Program Director (PD)/PI for a substantial NIH-independent research award.^6^ Early-stage investigator applications with scores within the funding range are prioritized for funding by the NIH. However, life- or work-related factors, e.g., fellowship training, childbirth, and relocation, fall within the early-stage investigator period for physician-scientists in neonatology. NIH allows early-career researchers to apply for an extension to their early-stage investigator status, which is considered on a case-by-case basis.

For this study, all new questions were pretested with a diverse convenience sample of NIH-funded United States neonatologists to assess face validity. Their pretest responses were not included in the final data set. The Baylor College of Medicine Institutional Review Board approved the study for IRB exemption.

Responses were analyzed using JMP 17.2.0 (SAS, Cary, NC, 2023). The analysis assessed the differences regarding scholarly accomplishments and career satisfaction between those who identify as men or women. As those who received international training also comprise a large portion of the research workforce, a comparison of international medical graduates (IMG) to American medical graduates (AMG) was performed. To explore the impact of racial and ethnic diversity, we compared those identifying as underrepresented in medicine (URIM), defined as those with identities of Hispanic, Latinx, Spanish, American Indian or Alaskan Native, Black or African American, or Native Hawaiian or other Pacific Islander, to those not commonly considered underrepresented in medicine. Respondents were also categorized into three groups to adjust for differences along career progression and seniority: early-career for those ≤10 years from fellowship completion, mid-career for those 11–20 years from fellowship, and late-career for those greater than 20 years out from fellowship. Comparisons between genders, medical graduate type, career phase, and intersection of gender and career phase were performed using the chi-square test for descriptive data, Kruskall Wallis test for non-parametric data, and two-way ANOVA for parametric distributions.

Multivariate analyses were conducted to assess factors that independently influenced select bivariate findings. All multivariate analyses started with the same covariate factors, including factors of personal characteristics, early career supports, clinical obligations, scholarly career accomplishments, and current organizational resources. From these collections of variables, all models were refined based on p-value and goodness of fit metrics (R2 adjusted or AUC) to optimize findings and minimize the risk of over-modeling. The total number of publications had a skewed distribution, so it was log-transformed for analysis, and multivariate analysis was performed using ordinary least squares. The percentage difference from the log-transformed ordinary least squares back converted to the number of publications using the median value of 45 total publications. The ordinal results for feelings of personal and professional balance, stress at work, and sadness or depression were converted into binary dependent variables for nominal logistic analysis, with results reported as odds ratios.

## Results

### Demographics

From this national survey of NIH-funded neonatologist physician-scientists (*n* = 131), 84 responded; 70 surveys had complete data (53% response rate) and were included in the analysis. As seen in Table 1, 47% identified as “man”, 53% as “woman”, and none as non-binary, prefer to self-describe, decline to respond, or other. Across career phases, 39% were categorized as early-career, 27% as mid-career, and 34% as late-career. Only 10% of respondents identified with racial or ethnic identities considered by the NIH to be underrepresented in medicine (African American or Black, American Indian and Alaskan Natives, Hispanic or Latinx or Spanish, or Native Hawaiians and other Pacific Islanders).^7^ Beyond their medical training, 34% held a master’s degree, and 17% held a PhD. Most respondents, 80%, were United States medical school graduates, but 1/5 were international medical graduates. Seventy-eight percent of early-career respondents were assistant professors, 58% of mid-career respondents were associate professors, and 92% of late-career respondents were full professors. Of these respondents, 61% were on tenure track. For an expanded description of demographic comparisons, see Supplemental Table S1.

**Table 1:** Participant Characteristics.

Year Completed Fellowship*	2008 (1995–2013)
Gender Identity**	
Man	33 (47%)
Woman	37 (53%)
Other	0 (0%)
Decline to Respond	0 (0%)
Race and Ethnicity**	
Hispanic/Latinx/Spanish	6 (9%)
American Indian or Alaskan Native	1 (1%)
Asian	13 (19%)
Black/African American	0 (0%)
Middle Eastern or North African	0 (0%)
Native Hawaiian or Other Pacific Islander	0 (0%)
White	53 (76%)
Other	2 (3%)
Sexual Identity**	
Lesbian or gay	0 (0%)
Straight, that is, not lesbian or gay	69 (99%)
Bisexual	0 (0%)
Another Description	0 (0%)
I don’t know	0 (0%)
Decline to respond	1 (1%)
Age*	46 (41–60)
Medical School Location**	
United States	56 (80%)
Caribbean	1 (1%)
Other	13 (19%)
Degrees Earned**	
MD/DO	70 (100%)
PhD	12 (17%)
MS	19 (27%)
MBA/MHA/MPH	5 (7%)
Other	0 (0%)

This table highlights the demographics of the NIH-funded neonatologists who responded to the survey. *n* = 70.

Results reported as:

*median (interquartile range).

**n (%).

### Scholarly support and productivity

Early career support came from varied sources, with 26% of respondents supported by an NIH training grant (T32), 24% received support from the NIH loan repayment program (LRP), 9% were trained under the Physician-Scientist Development Program (PSDP), and 51% did not receive research support from any of these mechanisms during training. When comparing genders across career stages, we found no differences in institutional support, type of research performed, tenure status, or distribution of their professional time dedicated to research during training. For international medical graduates (IMG), 92% received no research funding support from these mechanisms during training, compared to 42% of American medical graduates (AMG), *p* = 0.001.

NIH mentored career development awards (K awards) were received by 70% of respondents, with 63% of these as K08 awards (for basic science-oriented research) and 35% as K23 awards (for patient-oriented research). The median interval between the end of fellowship training and receipt of an NIH mentored award was four years (2 – 5 IQR). For an expanded description of early career support, see Supplemental Table S2.

The percentage of independent NIH research program (R-type award) investigators increased across the career spectrum (from early- to mid- and late-career). Among the mid- and late-career cohort, 56% (24 of 43 R-award recipients) had received both a mentored K- and independent R- series award. The median interval between the end of fellowship training and receipt of an independent research program (R-type) award was 10 years (7–12 IQR). The respondents classified their research type as basic (37%), clinical (30%), or translational (31%). Late-career investigators had more NIH programmatic (P-series: multi-project research programs) and cooperative agreement (U-series: high-priority research areas requiring substantial involvement from the NIH program or scientific staff) grants.

For non-NIH funding sources, 79% were supported by institutional grants, 49% by foundation grants, and 30% by industry funding. Across all three career phases, more men (45%) held industry grants than women (16%), *p* = 0.007, and most of these industry grants were held by those in the late-career stage, 12 of 21 (57%). Additional analysis of award history is available in Supplemental Table S3.

**Fig. 1:**
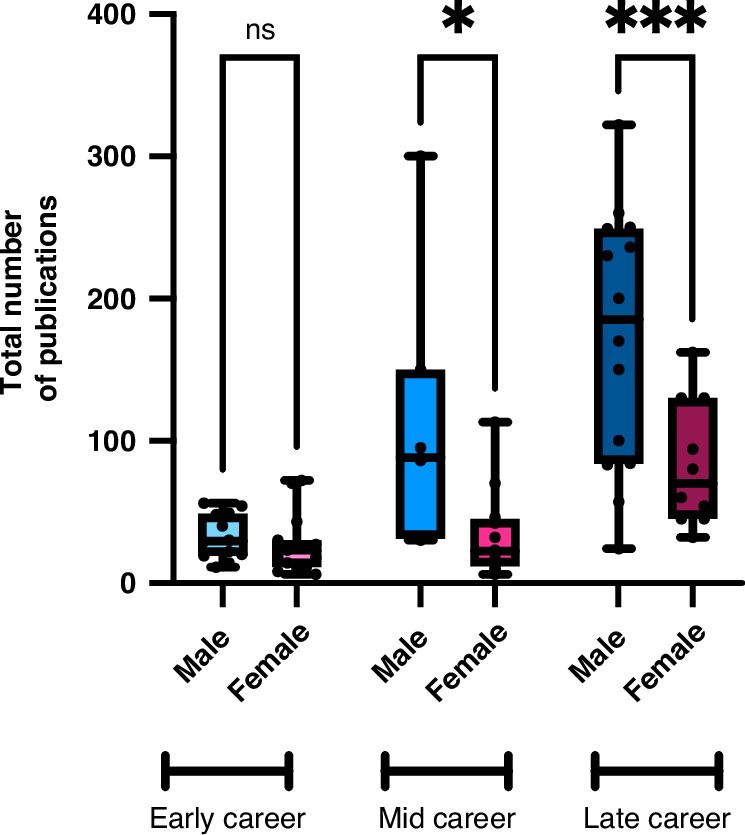
Total Number of publications by gender identity across career stages. This figure shows the total number of publications on the *y*-axis with career stage further divided by gender identity on *x*-axis with the results represented as box–whisker plots expressing median, and 10th, 25th, 75th, and 90th percentiles. Results compared using Kruskall–Wallis test with *p* < 0.05 determined as statistically significant. Range of *n* = 7–15.

We initially found a gender difference in the number of first or last authorships on manuscripts, where women averaged 17 such publications (11–35 IQR) and men 33 (15–55 IQR), *p* = 0.016; however, when adjusted for career stages, the statistical difference was lost. However, when comparing the total number of publications, the significant difference persisted. There was a significant difference in the reported total number of peer-reviewed publications between mid-career men with a median of 88 (31–150 IQR) authorships compared to women with 23 (12–45 IQR), *p* = 0.014. This disparity expanded further among late-career physician-scientists, where men had a median of 185 manuscripts (84–249 IQR) and women had 70 manuscripts (45–130 IQR), *p* = 0.014. See Fig. 1.

### Practice settings

For their first job after fellowship training, 87% of respondents started in an academic institution. In their first academic position, 25% (15 of 61) began as an instructor and 70% (43 of 61) as an assistant professor. Across identity groupings, only the comparison of those who identified as underrepresented in medicine (URIM) found a difference in appointed academic rank, with those not identifying as URIM more likely to receive the rank of Assistant Professor (75%) versus those identifying as URIM (33%, *p* = 0.001). When starting their first academic position, 83% of respondents reported ‘yes’ to having formal research expectations. An expectation for research productivity with their first job was significantly higher among late career women (10 of 10; 100%) compared to men (8 of 14; 62%, *p* = 0.026). 70% of respondents felt they had “protected” time for research during their first faculty position. Some of the clinical obligations during the first faculty position were greater among the late-career physicians who reported 16 (10–20 IQR) weeks of service, 60 (40–93 IQR) weeknight calls, compared to those early-career who reported 10 (8–14 IQR) weeks of service, *p* = 0.017, and 41 (23–51 IQR) weeknight calls, *p* = 0.013, or and mid-career individuals with and median of 10 (8–15 IQR) weeks of service, *p* = 0.028, and 40 (30–50 IQR) weeknights of call, p = 0.027 (See Supplemental Table S4 for more details). No differences were found in weekend coverage.

Only 10% of respondents started their first job after a fellowship with a funded NIH mentored K-type award. 54% (31 of 57) of respondents received institutional start-up funding support for their research. There was little variation in the duration of support for the start-up funds, with a median of 3 years (IQR 3 – 3). The median annual internal funding dollar amount was $50,000 ($28,750–$112,500 IQR). The start-up funds supported research supplies (49%), publications/meeting attendance (46%), research personnel (33%), and animal costs (33%). Please see Supplemental Table S2 for more details and comparisons.

Twenty percent applied to the NIH to extend their early-stage investigator status. Of those who applied for early-stage investigator extension, 7 of 14 (50%) were awarded an NIH independent research program R01-type award during this period, and more early-career women (6 of 15; 40% vs. 0 of 12 men) had applied for an early-stage investigator extension (*p* = 0.012).

All respondents felt their institution recognized and supported research as a critical mission. Recognition of physician-scientists by their institution for successful funding and high-impact publications was noted by 70% of respondents. This recognition was reported as praise (verbal, email, or local announcement) by 55%, monetary forms (bonus or promotion) by 23%, or a combination of these for 23%. When compared between genders, however, women were more likely to report recognition through praise (80%), and men via monetary rewards (40%), *p* = 0.004. To help achieve funding success, a formal institutional K-to-R mentorship program was present for 47%. Among other initiatives, 43% reported their institution had a formal program to link early-career physician-scientists across departments/divisions, 37% had a formal program to promote professional diversity within the research workforce, 46% had a formal mechanism for early-stage investigator to present their research and seek collaborators or mentors, and 67% had a formal mechanism to alert faculty to requests for proposals from industry, foundations, or other granting organizations.

Regarding the distribution of their current effort across different academic activities, 59% ( + /− 19% SD) of respondents’ time was dedicated to research, and 24% ( + /− 14% SD) of their time to patient care. The median number of weeks on clinical service was 8 (6–10 IQR), weeknight calls were 27 (12–45 IQR), and weekends covered were 14 (10–20 IQR). There were no differences found across career stages or genders, but those with international medical training worked more weeks of clinical service (10 versus 8, *p* = 0.022) and more weeknight calls (40 versus 24, *p* = 0.039), as compared to American medical graduates. See Supplemental Table S4.

### Mentorship

Eighty percent of respondents felt that mentors promoted them and their research. Of these respondents, 46% expressed that they benefited from mentorship received from internal mentors, while 33% felt that they benefited equally from internal and external mentors. No differences were seen in this sentiment across genders, career phases, or IMG status. See Supplemental Table S2 for additional details.

### Mentoring opportunities

Sixty-one percent of respondents reported having trainees engaged in their research before they obtained independent funding. Overall, the most common trainee groups were neonatology fellows (50%), followed by medical students (36%), pediatric residents (31%), and undergraduate students (29%). MD/PhD students were the least common trainees (7%). While having trainees involved in their mentored research was not found to be statistically different between groups, when exploring specific types of trainees, men (64%) were more likely to have neonatology fellows working with them than women (38%, *p* = 0.031). When looking across career phases, however, a gender difference was only found for those early in their career. Differences in trainee types were not found in other groups. See Supplemental Table S3.

The majority of respondents (73%) had trainees currently involved in their research. The median number of mentored trainees was 3 (2 -5 IQR). Neonatology fellows (56%) were again the most common trainee group, followed by undergraduate students (33%), medical students (29%), residents (24%), and post-docs (23%). The primary funding sources that supported trainee participation in their mentor’s research were grant funds (53%) and divisional/departmental funds (53%). For supporting research personnel, 84% reported using grant funds, and 54% also utilized divisional/departmental funds. See Supplemental Table S4 for current professional obligations, support, and accomplishments.

### Gaps in funding

39% reported funding gaps at some point during their career. The median duration of the funding gap was two years (1.5–3 IQR). During these gaps, research support was reported to come predominantly from internal funding resources (88%) or increased clinical time to support salaries (12%). Supplemental Table S3 includes additional details.

### Work-life interaction

**Fig. 2:**
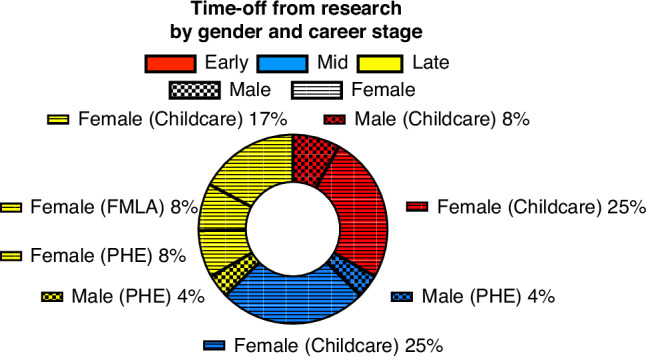
Comparison of types of leaves taken by gender identity across career stages. This pie chart demonstrates types of leave categorized as childcare Family Medical Leave Act (FMLA), and personal health emergency (PHE) by career phase (early, mid, late) and gender identity (male or female).

Eighty-nine percent of respondents had children; the median number of children per PI was 2 (2-3 IQR). At least one child was born during the early-stage investigator period for 77% of respondents. Of those with children, only 10% (6 of 62) reported that accommodations were made around childbirth. Of those 29% who took time off during their research career, it was most commonly for the birth of a child (75%), and was more likely to be reported by women, especially those later in their career. For those late-career, beyond childbirth, reasons for time off included family medical leave (25%) and personal health (25%). No difference was found for the number of individuals ever having taken time off across the career stages, but the number of time-off periods was significantly higher among those who were late-career women at 0 (0-2 IQR) vs. men with 0 (0-0 IQR), *p* = 0.039. See Fig. 2, and for more details Supplemental Table S5.

### Lessons from the COVID-19 pandemic

COVID-19 impacted work for nearly all investigators (96%), with 63% reporting temporary shutdowns of labs, 54% suspension of clinical studies, 53% delayed manuscript submissions, and 26% missed planned grant submission deadlines. International medical graduates were more likely to report being unable to complete a project (38% verse 7%, *p* = 0.002), that grant deadlines were missed (62% verse 18%, *p* < 0.001), and having had fewer opportunities for promotion (23% verse 2%, *p* = 0.002).

Though 90% reported working the same or more hours and 82% had unchanged patient encounters, 46% reported decreased compensation. Sixty-three percent reported spending more time with family, but many investigators experienced personal and family challenges. Negative impacts on personal life (childcare, teaching children, and stress with a spouse) and work-life balance were skewed towards early-career respondents and those underrepresented in medicine; however, no significant differences were reported by gender and IMG status. For further details on the impact of COVID-19, please see Supplemental Table S6.

### Career satisfaction

Overall, respondents expressed that if they had a choice, they would again choose medicine (99%), neonatology (93%), and being a physician-scientist (89%). Further, 81% plan to continue working as a physician-scientist over the next five years, but this rate decreased to 65% for late-career neonatologists. No gender differences were found for this measure across the three career stages.

**Fig. 3:**
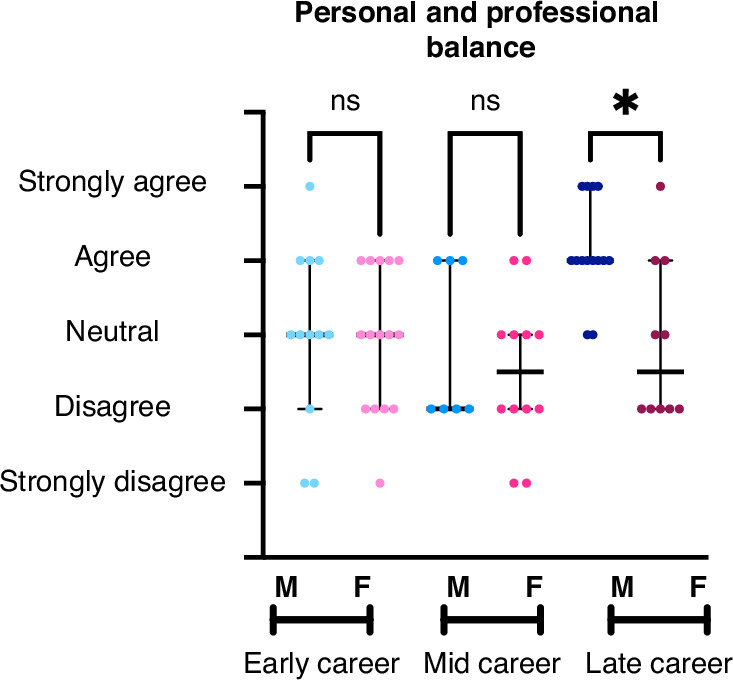
Reported agreement with the statement “Feelings of Balance Between Personal and Professional Commitments is about right” by gender identity across career stages. This figure shows a 5-point Likert scale ranging from Strongly Agree to Strongly Disagree on the *y*-axis with career stage further divided by gender identity on *x*-axis. Results show as median with 95% confidence interval. Results compared using Kruskall–Wallis test with *p* < 0.05 determined as statistically significant. Range of *n* = 7–15.

**Fig. 4:**
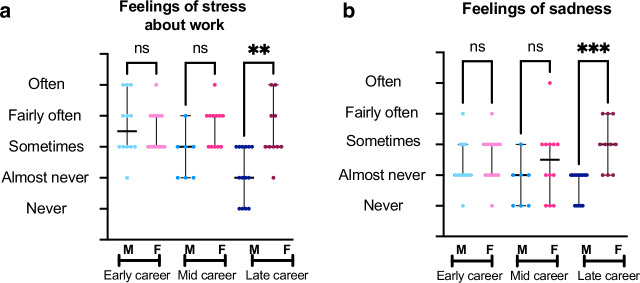
Reported feelings of stress and feelings of sadness and depression by gender identity across career stages. **a** Reported Agreement with the statement “How Often in the Past Year Respondents Felt Stressed about Work” by Gender Identity across Career Stages.  This figure shows a 5-point Likert-scale ranging from Never to Often on the *y*-axis with career stage further divided by gender identity on x-axis. Results show as median with 95% confidence interval.  Results compared using Kruskall–Wallis test with *p* < 0.05 determined as statistically significant. Range of *n* = 7–15.  **b** Reported Agreement with the Statement “How Often in the Past Year Respondents Felt Sad or Depressed” by Gender Identity across Career Stages.  This figure shows a 5-point Likert-scale ranging from Never to Often on the y-axis with career stage further divided by gender identity on *x*-axis. Results show as median with 95% confidence interval.  Results compared using Kruskall–Wallis test with *p* < 0.05 determined as statistically significant. Range of *n* = 7–15.

Regarding the statement that “the balance between personal and professional commitments was about right”, late-career men were more likely to report in the affirmative than women (Fig. 3, *p* = 0.007). No differences were found in agreement with this statement for those in the early- and mid-career stages. While physician-scientist men reported rarely feeling stressed about work, mid-career women reported feeling stressed more often (*p* = 0.033). Among late-career respondents, women were more likely to report feeling stressed about work than men, p = 0.014 (Fig. 4a). Early career physicians did not report significant differences between gender identities in terms of feelings of work stress. Late-career women were more likely to report “Sometimes” having feelings of sadness or depression over the past year, compared to late-career men who were more likely to report “Almost Never” having such feelings, *p* < 0.001 (Fig. 4b). No gender differences were identified for those early- or mid-career. For more results on career satisfaction, please see Supplemental Table S7.

### Regression adjusted associations

We constructed multivariate models to understand better how multiple factors may interact and impact identified results for total publications, feelings of personal and professional balance, feelings of stress about work, and feelings of sadness.

**Fig. 5:**
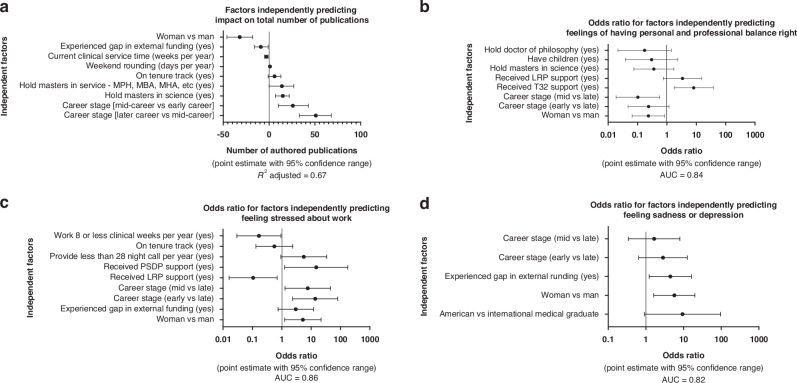
Multivariate analysis of independent impacts of factors contributing to total publications, personal and professional balance, feelings of stress about work, and feelings of sadness. **a** Total publications as predicted by ordinary least squares with the percentage difference from the log-transformed ordinary least squares back converted to the number of publications using the median value of 45 total publications to report median point estimate with 95% confidence range. *R*^2^ adjusted = 0.67. **b** Personal and professional balance as predicted by nominal logistic regression to report odds ratios with 95% confidence range. AUC = 0.84  **c** Feelings of stress about work as predicted by nominal logistic regression to report odds ratios with 95% confidence range. AUC = 0.86. **d** Feelings of sadness as predicted by nominal logistic regression to report odds ratios with 95% confidence range. AUC = 0.82.

Multivariate analysis of factors contributing to the number of publications produced by respondents found that career stage was the strongest positive predictor of publications. On average, being a woman predicted 32 fewer publications, *p* < 0.001, see Fig. 5a. For feelings of personal and professional balance, career stage and gender were powerful predictors in the model. Being mid-career, compared to late-career, had a lower odds ratio at 0.10 (*p* = 0.009) for agreeing or strongly agreeing that personal and professional balance was right, see Fig. 5b. Similarly, being a woman had a lower odds ratio of 0.23 (*p* = 0.026) than a man.

Regarding feeling stressed about work, career stage and gender remained significant independent predictors, with an odds ratio over 5 (*p* < 0.05), see Fig. 5c. In the model of feeling sad or depressed, identifying as a woman was the most significant factor with an odds ratio of 5.7 (p = 0.007) as compared to a man, see Fig. 5d. For numeric results of multivariate analysis, please see Supplemental Table S8.

## Discussion

Physician-scientists are uniquely positioned to contribute to medical and scientific discovery, which is critical to the evolution of the practice of medicine and human health. With fewer physicians pursuing careers as physician-scientists, there is a growing need to identify the barriers to this career path and key supports for research success.^2^ We describe several factors related to demographics, institutional support, scholarly productivity, funding trajectory, work-life balance, and career fulfillment among physician-scientists in neonatal-perinatal medicine across career stages, gender identity, IMG status, and racial and ethnic identity. Further, we identified notable differences between men and women, AMG and IMG status, and career stages among neonatologist physician-scientists.

The demographic factors highlight a few interesting facts about this cohort of NIH-funded physician-scientists in neonatal-perinatal medicine. Twenty percent of the cohort had IMG status; the majority were MD-only applicants. IMGs constituted 38% of the neonatology workforce in 2020.^8^ IMGs have become an increasingly important part of the biomedical research workforce in the United States, particularly in neonatology. Between 1984 and 2004, the number of IMG full-time faculty more than doubled, as reported by the Association of American Medical Colleges and the NIH. Additionally, the representation of IMGs as principal investigators (PIs) on NIH research grants rose from 16.5% to 21.3% over the same period, similar to the representation found in this study.^9^ IMGs filled 20% of neonatology fellowship positions in the 2024 appointment year, as reported by the National Resident Matching Program (NRMP) data. Complicating the research careers for IMGs working in the United States on a visa are barriers to eligibility for many federal funding mechanisms, including participation in NIH-funded research training grants. Due to visa-related constraints, such barriers may hinder international physician-scientists’ research trajectory.^10^

Pediatric training programs are often restructured to aid the career trajectories of MD-PhD candidates who are an asset to the physician-scientist workforce. Still, trainees who embark on the research career path later (MD-only) during their training were also successfully funded, as shown by the study results. Ensuring this pool of researchers also receives support to promote their success as researchers will be critical to maximizing scientific advancement in the field. A recent report from the NIH noted that from 2014-2023, the proportion of applications by and awards to PIs with single medical degrees trended down, while the proportion of applications from and awards to PIs in the dual degree and “other” degree categories increased.^11^

Early financial support is critical to success as a physician-scientist. The NIH Loan Repayment Programs (LRPs) are critically crucial for physician-scientists in neonatology, as they address one of the significant barriers to pursuing a research-intensive career: educational debt. The high cost of medical education often deters physicians from engaging in research, as they may prioritize higher-paying clinical positions to manage their debt. The LRP provides financial relief by repaying a portion of the educational loans, enabling physicians to focus on research with a reduced debt burden.^12^ About a quarter of the respondents in this study benefited from the LRP (https://www.lrp.nih.gov/). Institutional NIH training grants (ie. T32) are also an important initiative to support the budding physician-scientist, providing salary support and protected time for their development.^2^ Selection into and participation in an NIH institutional training grant provides comprehensive research training, increases the likelihood of academic appointments, and offers supplemental mentorship and networking opportunities. An NIH-funded institutional training grant supported more than a quarter of this cohort, highlighting its importance. This has to be noted in contrast to the 92% of NIH-funded IMGs who never received this support.

Our study did not find significant differences between genders in receipt of K or R awards or time from K to R awards. Similarly, K-to-R transition rates by gender were not different within pediatrics departments, according to a study by Nguyen et al.^13^

This study revealed that despite no gender-based differences in first- and last-author publications, there was a significant decrease in total publications from mid- and late-career physician-scientist women compared to men colleagues. As publications and collaborations can significantly impact grant funding, mentorship, networking, and future successes, these differences may impact future professional accomplishments and career satisfaction or reflect past career experiences and inequities among women. Significant disparities between the number of scientific works men and women produced have been well-documented.^14^ This gap may stem from differences in productivity due to systemic reasons or from the lack of recognition of the contributions of women. Ross et al. reported that part of this gap results from unacknowledged contributions by women scientists.^15^ Co-authored publications, as part of a team science approach, are critical and contribute to the overall productivity of a physician-scientist. Further research into the reasons behind this discrepancy may help to address this inequity.

Implementing standardized guidelines for physician-scientist support may lead to greater uniformity across institutions. A national focus group, workshop, and survey engaging junior and senior academicians collected recommendations on the ideal levels of protected time and resources necessary to launch independent research.^16,17^ Their findings suggested that early-career faculty should be limited to 8 weeks of clinical duties, no more than 24 night calls per year, and provided start-up funds between $500 K and $1 M with a guarantee of 5 years of salary support. The numbers reported among early-career respondents in our cohort were 25% higher for the clinical time, 10-20% of the start-up fund value, and only 60% of the recommended start-up duration. This may contribute to the lower percentage of neonatologists engaged in research than other pediatric subspecialists.

The high levels of reported career satisfaction with being a neonatologist physician-scientist are inspiring. Consistent with other findings in pediatrics, the higher rates of feelings of work stress and feelings of sadness among women in this study highlight that even the draws of scientific discovery do not lessen these concerns.^18^ Others have explored potential contributing factors to these findings, and recommendations for women in medicine have been published.^19–21^ Ultimately, these findings and the recommendations can be distilled to recognition of and action to address implicit biases, organizational transparency with professional obligations, promotion, compensation, and equity of professional and personal time. To improve recruitment, retention, and career satisfaction in the physician-scientist pathway, equity and quality of time need to be addressed, which is especially crucial in the context of a growing predominance of women in the field. Societal and institutional support for men to take time off needs to be popularized and actions taken, so men can more easily share in domestic burdens and their professional impact. For women, optimal resources to support their professional and domestic obligations need further study and advocacy. When examining these effects in the context of the neonatologist physician-scientist, small differences may be amplified over time or reflect the disadvantaged experiences of years gone by. But without equity in the early-career stages, it is a reasonable certainty that these inequities will only widen as one’s lived experiences progress.

As others have discussed, the COVID-19 pandemic significantly impacted the productivity, discovery, and resiliency of physician-scientists and their research.^22^ Our findings support these previous studies. The COVID-19 pandemic exposed vulnerabilities within our healthcare system. It highlighted that investment and systematic changes are needed,^23^ including mentorship, support through gaps in funding and productivity, and matching researchers with institutions that will best help them. At its core, as Oishi et al. espouse, institutions must put their faculty’s needs ahead of the division’s.^17^ Investment in human capital will have substantial long-term returns on productivity and discovery.

Limitations of this study include a small cohort that may impact the ability to determine statistical and practical differences between the groups compared. Further, these data were collected by anonymous self-report, which precludes data validation, further exploration, or clarification of the obtained results. The reported results are also at risk of recall bias, which is further compounded as many of the questions requested answers may be more recent for some respondents and more distant for others. Additionally, by sampling only one year of currently NIH-funded researchers, survivor bias may confound this study. Future work to explore multiple years of currently, consistently, or previously NIH-funded researchers might better address this concern.

The strengths of this study include a high response rate and a focus on neonatal-perinatal medicine, career stage, medical graduate status, and gender-based factors. The methodology also affords participants anonymity for honest responses, fostering highly granular data from questions with a high degree of face validity and comparison across other studies. Further, using multivariate analysis allowed for further assessment of the independent impact of individual factors to correct for confounding.

A dwindling physician-scientist workforce is a challenge that all pediatric subspecialties face, including neonatal-perinatal medicine. We have highlighted positive factors and gender- and career-based factors that require attention. To attract, develop, and retain more physician-scientists, training programs and institutions must implement strategies that motivate talented neonatologists to pursue the physician-scientist career pathway.

## Conclusion

From the responses of this cross-sectional sample of NIH-funded neonatologist physician-scientists, we found commonalities and differences across career phases, gender, IMG status, and among racial and ethnic identities. There is a universal need for improved support for early-career investigators, financial and logistical support during gaps in funding, and protected time to meet the professional expectations of physician-scientists. Differences persist in periods of time-off, scholarly productivity, funding sources, and ultimate career satisfaction. As policymakers and professional societies seek to improve the pathway into a career as a physician-scientist, consideration of these financial and human factors will help craft holistic recommendations and increase the number of those entering this important career path.

## Supplementary information

Supplemental Table S1.

Supplemental Table S2.

Supplemental Table S3.

Supplemental Table S4.

Supplemental Table S5.

Supplemental Table S6.

Supplemental Table S7.

Supplemental Table S8.

Suppemental Table 9.

## Data Availability

Please see interactive data visualizations offered in Supplemental Table S9. The dataset generated and analyzed during the current study are available from the corresponding author on reasonable request.
