# Supplementary material for: NIH-funded neonatologist physician-scientists: an exploration of equity and success
Source: Pediatr Res. 2025 Jun 30;99(2):573–80. doi: 10.1038/s41390-025-04224-5 (PMC12956553; doi:10.1038/s41390-025-04224-5)
Supplement: Supplementary file 9 — Suppemental Table 9. [file 41390_2025_4224_MOESM9_ESM.docx]

Supplemental Table S9: Interactive Data Visualizations to Explore the Collected Data for Additional Insights

| Respondent Characteristics | <https://public.tableau.com/app/profile/eric.horowitz/viz/20250213NIHNeoPSTableau-Characteristics/Characteristics> |
| --- | --- |
| Summary of First Job after Fellowship | <https://public.tableau.com/app/profile/eric.horowitz/viz/20250213NIHNeoPSTableau-1stjob/1stJob> |
| Award History | <https://public.tableau.com/app/profile/eric.horowitz/viz/20250213NIHNeoPSTableau-awardhx/AwardHx> |
| Accommodations to Promote Early Career Success | <https://public.tableau.com/app/profile/eric.horowitz/viz/20250213NIHNeoPSTableau-Accomodations/Accomodations> |
| Supports for Personal Demands | <https://public.tableau.com/app/profile/eric.horowitz/viz/20250213NIHNeoPSTableau-Support/Support> |
| Current Professional Duties | <https://public.tableau.com/app/profile/eric.horowitz/viz/20250213NIHNeoPSTableau-Currentduties/CurrentProfessional> |
| Current Research Activities and Supports | <https://public.tableau.com/app/profile/eric.horowitz/viz/20250213NIHNeoPSTableau-Currentresearch/CurrentResearch> |
| Impact of COVID on Research, Career, and Personal Life | <https://public.tableau.com/app/profile/eric.horowitz/viz/20250213NIHNeoPSTableau-COVID/COVID> |
| Career Satisfaction | <https://public.tableau.com/app/profile/eric.horowitz/viz/20250213NIHNeoPSTableau-Satisfaction/careerSatisfaction> |
